# Use of social deprivation status in primary prevention cardiovascular risk scores: a must but a challenge

**DOI:** 10.1093/postmj/qgae043

**Published:** 2024-03-28

**Authors:** Dorien M Kimenai, Anoop S V Shah

**Affiliations:** BHF Centre for Cardiovascular Science, University of Edinburgh, Edinburgh EH16 4SA, United Kingdom; Department of Non-Communicable Disease Epidemiology, London School of Hygiene & Tropical Medicine, London WC1E 7HT, United Kingdom

**Keywords:** social class, cardiovascular diseases, risk prediction, general population

## Association between social deprivation and cardiovascular disease

Addressing health inequalities is a global priority. When focusing on poverty, it has become clear that a low social deprivation status is strongly associated with increased risk of cardiovascular disease [1]. Although there is no global definition for social deprivation, income, education, crime and employment are fundamental domains that are used to define the deprivation status of an individual. Social deprivation is often an area-based metric and characterizes an individual’s social deprivation status for a specific region using these multiple domains. People living in more deprived areas experience more cardiovascular events and die earlier compared to those who are living in more affluent areas. These observations are evident across all age groups [2, 3]. The underlying reasons for the association between increased social deprivation and cardiovascular disease are complex and multifactorial. However, incorporation of social deprivation status to risk estimation systems to better target preventative strategies is likely to hold major promise and ultimately reduce the existing inequality gap.

Cardiovascular risk estimation systems are a key component in the detection of high risk individuals with better targeted therapy to prevent future cardiovascular disease. In this editorial, we provide an overview on the evidence of using clinically applied primary cardiovascular risk scores in relation to social deprivation.

## Use of social deprivation in clinically applied cardiovascular risk scores

Primary prevention cardiovascular risk scores are tailored for specific regions and countries to ensure we are making an accurate cardiovascular risk estimation for the population of interest. As such multiple cardiovascular risk scores have been developed and validated for use in clinical practice which are region and, in some cases, population specific [4–7]. The use of social deprivation as a variable in primary prevention cardiovascular risk estimation systems is not ubiquitous and region-dependent. Omission of deprivation status from cardiovascular risk estimation system may further increase the current inequality gap, given the known association between affluence and cardiovascular disease. We recently compared ASSIGN (Assessing cardiovascular risk using SIGN [Scottish Intercollegiate Guidelines Network] guidelines to ASSIGN preventive treatment), a risk estimation system used in Scotland that includes social deprivation as a covariate in the risk score, to Europe-based Systematic Coronary Risk Evaluation 2 algorithm (SCORE2) and USA-based Pooled Cohort Equations (PCE) that do not account for deprivation status [8]. We assessed these three clinically applied primary prevention cardiovascular risk scores across different groups of social deprivation on the performance of predicting future cardiovascular events. We found that the two risk scores that do not incorporate social deprivation into the risk equation—SCORE2 and PCE—persistently underestimated cardiovascular risk in those who are living in the most deprived areas. In contrast, ASSIGN, that does include social deprivation, performed well across all groups of social deprivation. This observation was recently confirmed in a Dutch study authored by Kist and colleagues demonstrating a poorer performance of SCORE2 in individuals those who were most deprived [9]. This analysis provides further growing evidence for the important role of deprivation status in improving cardiovascular risk estimation systems, especially when applied to individuals who are less affluent.

## Challenges to incorporate social deprivation into cardiovascular risk scores

A common approach to incorporate social deprivation is by developing and using deprivation status based on area or postal codes. For example, the ASSIGN risk score uses the Scottish Index of Multiple Deprivation score to identify an individual’s social deprivation status. The Scottish Index of Multiple Deprivation score is an area-based metric that is derived from individuals’ postal codes and compiled using seven domains of deprivation (income, employment, education, health, access to services, crime, and housing). Cardiovascular risk scores that are country-specific—like ASSIGN and QRisk3—have the advantage that they can easily incorporate a nationally available measure of social deprivation.^5 6^ It becomes more challenging for cardiovascular risk scores that goes beyond borders, like SCORE2. As SCORE2 is developed and validated in a large number of cohorts across Europe [4], there is a lack of a uniform measure of social deprivation and therefore difficult to create a cardiovascular risk score that includes social deprivation as a covariate into the risk equation. A solution might be for SCORE2 to add an additional recalibration step at the country level for social deprivation status, to allow more precise risk estimation.

Several further issues also remain pertinent to area-based metrics of social deprivation. First, it is also important to mention that commonly applied risk scores that do include social deprivation status as risk factor uses an indirect area-based measure of social deprivation. Discordance between area-based deprivation and individual degree of affluence is likely to be substantial [10]. Second, social deprivation is a composite of multiple domains (e.g. income, education, crime and employment), and it is not well established which individual domains are particularly important for an accurate prediction of someone individual’s cardiovascular risk. Third, deprivation is dynamic with areas becoming more or less affluent. This means an additional practical step of updating risk estimation systems at regular intervals with both time and resource implications is required [6].

## Future perspectives

In our goal to optimally prevent cardiovascular disease across the globe, it is important that cardiovascular risk estimation systems strongly consider the role of social deprivation as a covariate. While underlying causes that result in the worse outcome for those who are living in more deprived areas are complex, detecting at-risk populations will allow health systems and health policy strategies to better target the population and ultimately reduce the cardiovascular morbidity and mortality.
